# Rare Case of Primary Bone Lymphoma

**DOI:** 10.7759/cureus.84415

**Published:** 2025-05-19

**Authors:** Madeline L Fudala, Imad Karam, Aye M Thida, Imran Khan, Rachelle Hamadi, Natasha Macapagal-Brown, Raavi Gupta, Mohan Preet

**Affiliations:** 1 Hematology and Oncology, State University of New York, Downstate Health Sciences University, Brooklyn, USA; 2 Pathology, State University of New York, Downstate Health Sciences University, Brooklyn, USA

**Keywords:** cutaneous involvement, diffuse large b lymphoma, extranodal diffuse large b-cell lymphoma, primary bone lymphoma, primary diffuse large b-cell lymphoma, rare lymphoma

## Abstract

Primary bone lymphoma (PBL) is a rare entity defined as a lymphoid neoplasm of bone that occurs in the bone without involvement of lymph nodes or other extranodal sites. In this report, we present a case of an elderly female with PBL who presented with regional lymphadenopathy and cutaneous lesions mimicking the primary cutaneous diffuse B-cell lymphoma (PCDBCL), leg type.

This review describes the case of a 73-year-old female with primary bone lymphoma who presented with cutaneous lesions and regional lymphadenopathy. She was initially diagnosed with Paget’s disease of bone but ultimately presented to the emergency room with a pathologic fracture of the left tibia and painful overlying cutaneous lesions. Imaging studies, including X-ray and computed tomography (CT) scans, along with a tibial biopsy, revealed findings consistent with PBL, characterized by large lymphoid cells that were positive for CD10, CD20, and Bcl6, with a high Ki-67 index. The diagnosis was confirmed despite the atypical cutaneous involvement. Due to her age, low-performance status, and extranodal disease, the patient was classified as high-intermediate risk according to the National Comprehensive Cancer Network International Prognostic Index (NCCN-IPI) criteria. The presence of extranodal involvement also placed her at intermediate risk for central nervous system (CNS) disease.

The clinical presentation of PBLs is often nonspecific, and diagnostic criteria noted in the literature vary, making for a difficult diagnosis in clinical practice. To our knowledge, this patient is the first documented case of PBL with concurrent cutaneous manifestations and regional lymph node involvement.

This case report and literature review investigate the possibility of PBLs presenting with lesions extending beyond the initial area of bony involvement.

## Introduction

Primary bone lymphoma (PBL) represents less than 5% of all primary bone tumors [1]. Due to the rare incidence, nonspecific initial presenting clinical features, and other more common differential diagnoses with similar presentation make for a difficult diagnosis [2]. The 2020 World Health Organization classification of soft tissue and bone tumors defines PBL as a lymphoid neoplasm presenting with lesions exclusively of bony origin without nodal or extranodal involvement [2,3]. Based on the literature review, most patients presented vaguely with symptoms of localized pain, swelling, and pathologic fracture, with the femur and pelvis being commonly involved [2,4-10]. Although less frequent, other literature has presented cases of PBL with bone lesions similar to Paget’s disease or osteomyelitis [11,12]. To our knowledge, PBL presenting as a combination of tibial involvement and cutaneous lesions mimicking primary cutaneous diffuse large B-cell lymphoma (PCDBCL), leg type, has never been reported.

## Case presentation

**Figure 1:**
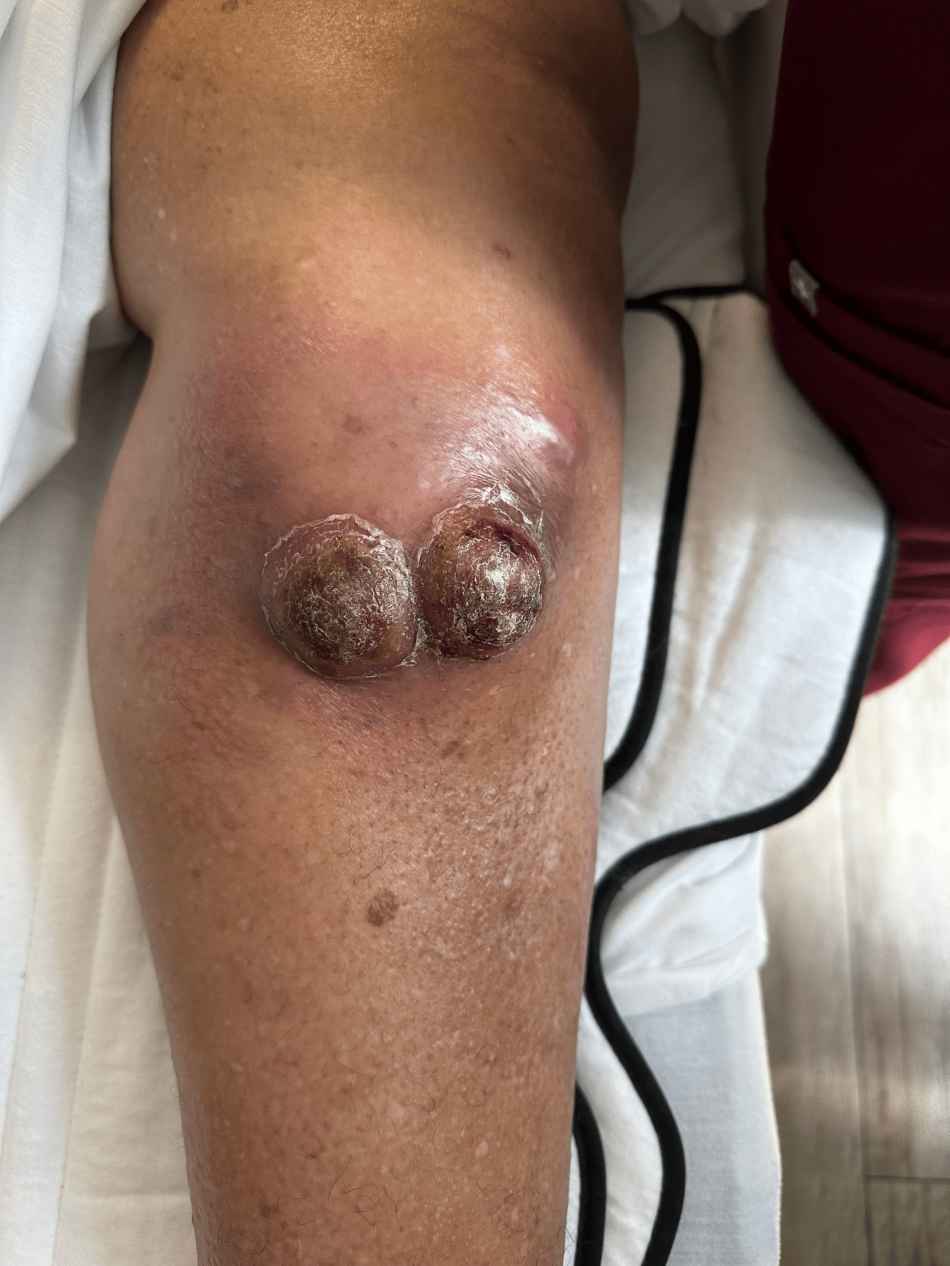
Nodular cutaneous lesions overlying the right proximal tibia

This is a 73-year-old female with a medical history significant for hypertension, hyperlipidemia, gastroesophageal reflux disease, type 2 diabetes mellitus, paroxysmal atrial fibrillation, heart failure with reduced ejection fraction, and automatic implantable cardioverter defibrillator placement. She presented to the emergency room due to increasingly painful, atraumatic left lower extremity pain. Plain film X-rays of the left leg reported bone lesions initially thought to be Paget’s disease. Shortly thereafter, the patient began to develop swelling, erythema, and cutaneous masses. These masses were initially small, soft, cutaneous, and tender but later enlarged to form two distinct, adjacent, hardened, and scaly lesions. They developed surrounding swelling and erythema and grew to occupy an area of 12 cm over the patient’s left proximal tibia (Figure 1).

**Figure 2:**
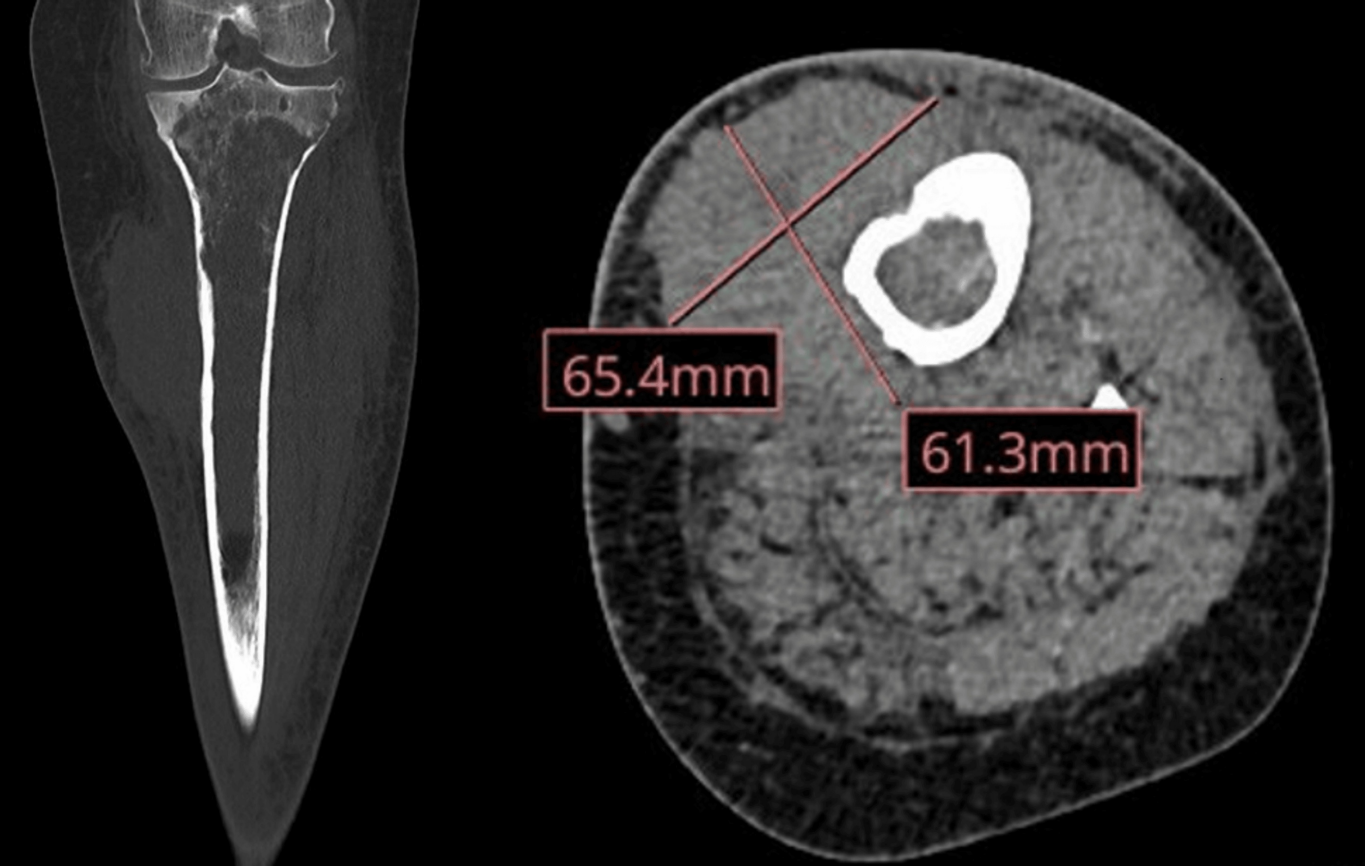
CT right proximal tibia

A CT scan with intravenous iodinated contrast of the left lower extremity revealed a proximal tibial lesion with marrow replacement and multifocal cortical breaks. In addition, there was an extraosseous enhancing mass along the left tibia with significant soft tissue infiltration (Figure 2). This was followed by a bone 18F-sodium fluoride positron emission tomography scan (NaF PET), which showed a destructive lesion of the left tibia with an associated soft tissue lesion. Enlarged left femoral lymphadenopathy consistent with metastatic nodal disease was also present. 

**Figure 3:**
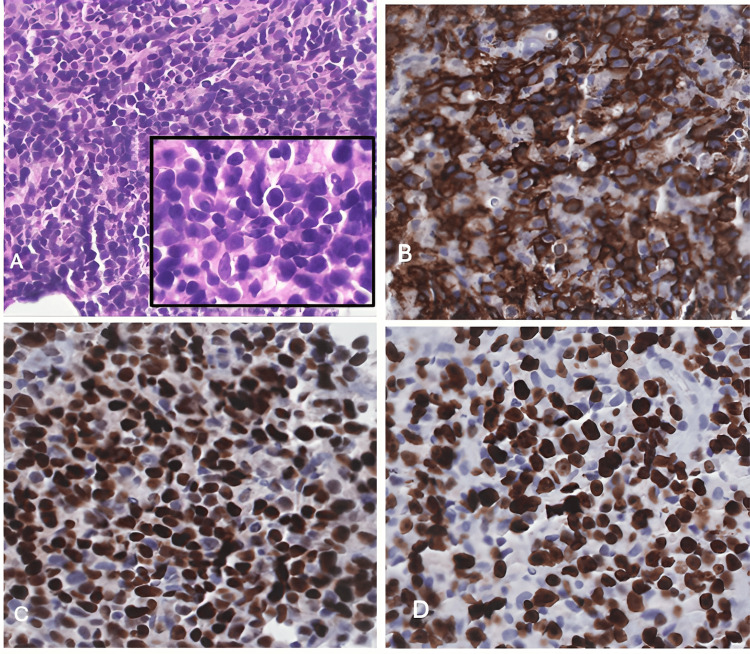
Bone core biopsy A: The tibial bone core biopsy shows a sheet of large lymphoid cells with a background of few small lymphoid cells. B: The large cells are positive for CD20. C: positive for BCL6 and CD10 and negative for BCL2. D: The lymphoma cells show a high Ki-67, proliferation index (85%).

The tibial bone core biopsy showed a sheet of large lymphoid cells with a background of a few small lymphoid cells (Figure 3A). The large cells are positive for CD20 (Figure 3B), BCL6 (Figure 3C), and CD10 and negative for BCL2. The lymphoma cells show a high Ki-67 (Figure 3D) proliferation index (85%). The presence of a primary 4cm tibial mass with X-ray features of permeative moth-eaten marrow and histologically presenting as a diffuse large B cell lymphoma with germinal center cell phenotype was consistent with primary bone lymphoma. Lymph node involvement was suspected to be a consequence of the extension of the lymphoma.

## Discussion

PBL is a rare malignant neoplasm accounting for only 2-5% of all malignant bone tumors [1,4-6,13]. Despite its rarity, PBL is associated with a favorable prognosis compared to other primary bone tumors and lymphomas with secondary involvement of the bone [1,2,4,6]. However, the infrequency of cases makes for a challenging diagnosis due to the lack of clearly elucidated clinical features and controversies among the definitions of what constitutes a PBL diagnosis. Some literature proposes that the average time from symptom onset to diagnosis may be as long as eight months [2]. This was evident in our patient, who experienced a six-month delay between her initial presentation and final diagnosis.

The 5th edition of the World Health Organization has defined PBL as a lymphoid neoplasm of bone with no extranodal or visceral involvement for at least six months from the time of initial diagnosis [3]. However, other, broader definitions of PBL are also present in the literature. For example, Heyning et al.’s definition permits regional lymph node involvement at the time of diagnosis if no features suggesting earlier lymphomatous involvement are present elsewhere in the body [7]. Other definitions are even more all-encompassing and, instead, stratify based on regional lymph node involvement and the presence of visceral disease [4].

Clinical presentations of PBL vary, are nonspecific, and tend to mimic other diseases. Imaging studies of PBL closely resemble those of secondary bone lymphomas, making them increasingly difficult to distinguish clinically. However, most of the existing literature agrees that the most common presenting symptoms are pain, palpable masses, and pathologic fractures [1,2,4-6,11,14]. Notably, classic “B” symptoms seem absent in most patients, especially early in the disease course [2,4-6]. This was consistent with our case, as our patient’s primary presenting symptom was pain.

Many imaging modalities have been used to assist with diagnosis, biopsy guidance, and monitoring response to treatment. Plain X-rays are commonly used initially for suspected cases of PBL, but presenting features range from no apparent radiographic changes to a permeative “moth-eaten” pattern of bone destruction [1,2,5,6,11]. Magnetic resonance imaging (MRI) has recently been used to detect PBL earlier due to its superior ability to visualize bone and soft tissue involvement [2]. Additionally, positron emission tomography-computed tomography (PET-CT) is increasingly utilized to assess the functional characteristics of masses [1,2]. It has been proposed that advancements in imaging have contributed to the controversy surrounding the definition of PBL due to the improved ability to visualize disease in other body regions outside the primary bone lesion [2,13].

Our patient is the first documented case, to our knowledge, of primary bone lymphoma (PBL) presenting with concurrent cutaneous manifestations and regional lymphatic spread. The initial imaging revealed a mass resembling Paget’s disease, which contributed to the delay in diagnosis. Furthermore, while MRI could have facilitated a more precise characterization of the lesions at an earlier stage, it could not be performed due to the incompatibility of the patient's cardiac pacemaker. The severe cutaneous lesions at the time of her most recent hospitalization posed a diagnostic challenge, as it was clinically difficult to distinguish between a primary bone lymphoma that had invaded the overlying skin and a primary cutaneous lymphoma that had metastasized to adjacent bone. After an extensive chart review and consulting with orthopedics and pathology, malignancy was suspected to be a PBL that was present at the time of symptom onset in early 2024. This case report emphasizes the importance of considering PBL even in patients with atypical presentations due to inconsistencies in diagnostic criteria for the disease.

Despite the diagnostic challenges, treatment of PBL tends to be similar to any other diffuse large B-cell lymphoma (DLBCL). The literature describes a preference for chemotherapy with R-CHOP (rituximab, cyclophosphamide, doxorubicin, vincristine, prednisone) +/- local radiation [2,5,10,13].

## Conclusions

In summary, we present the case of an elderly patient with an atypical presentation of PBL, initially misdiagnosed as Paget’s disease based on radiographic and physical examination findings. Follow-up imaging and biopsy ultimately confirmed the diagnosis of PBL, leading to the development of an appropriate management plan. Of significance, the combination of diagnostic inertia and poorly elucidated presenting features of PBL contributed to the challenges in reaching a timely diagnosis. This case led us to attempt to elucidate the clinical manifestations of this rare malignancy and explore the possibility of extra-bony lesions at the time of presentation. A high clinical suspicion for this rare diagnosis is required for prompt diagnosis and appropriate management.
